# COVID-19: New adaptation for IVF laboratory protocols

**DOI:** 10.5935/1518-0557.20200054

**Published:** 2020

**Authors:** Syed Waseem Andrabi, Mir Jaffar, Puneet Rana Arora

**Affiliations:** 1 Division of Embryology, Milann-The Fertility Centre, New Delhi, India; 2 Division of Embryology, Milann-The Fertility Centre, Bangalore, India; 3 Clinical Division, Milann-The Fertility Centre, New Delhi India

**Keywords:** COVID-19, IVF laboratory, new adaptations

## Abstract

Severe acute respiratory syndrome coronavirus-2 (SARS-CoV-2) widely known as COVID-19 was first reported in late December 2019, in China. Since then this novel coronavirus has spread throughout the world. Our return to normal life will not take long, for we are in a phase where the COVID-19 curve is stabilizing. ART services must return to operation, since infertility is also a disease and treatment has to start. Before resuming ART treatments, it is very important to consider local and national regulations. Change is mandatory, to set us back to successful ART treatment without compromising on quality, and to minimize the spread of COVID-19 among staff and patients; and for this we need to take measured and vigilant steps.

## COVID-19 Transmission

 Like the previous two varieties, SARS and MERS, responsible for the previous two major respiratory disease outbreaks, COVID-19 shows a marked predisposition for the alveolar epithelial cells of the human lung, using angiotensin-converting enzyme 2 (ACE2) as its entry point (Zhou *et al*., 2020), unlike its two major predecessors, which could penetrate cells via other routes as well. Consequently, COVID-19 cannot enter cells that do not carry ACE2 on the surface. ACE2 was reportedly detected in Leydig and Sertoli cells, and in spermatogonia of the human testis, theca, and granulosa cells of human ovaries (Zhou *et al*., 2020). Mature human spermatozoa and oocytes are devoid of ACE2 receptors. There are no reports of vertical disease transmission from parents to children concerning the previous SARS outbreak, in 2002-2003, which also uses ACE2 as the main point of entry into cells (Schwartz & Graham, 2020). There is much controversy concerning the vertical transmissions of COVID-19 to infants before birth from an infected mother. In a study involving 9 infants, the authors reported no transmission from mother to child (Chen *et al*., 2020); notwithstanding, another report mentioned a newborn child with elevated IgM antibodies and abnormal cytokine results, two hours after birth (Dong *et al*., 2020), although the possibility of infection during delivery cannot be rule out in the latter case.

## Positive Hope

Viral presence in the reproductive tract in males highly increases the risk of sexually transmitted infections. It can also affect the fertility in males by infecting locally or through spermatogonial stem cells. Although it is not clear to what extent the virus exists or replicates in the semen (Mansuy *et al*., 2016), some viruses - like the Zika virus, can live up to one year after recovery in males (Kurscheidt *et al*., 2019). To date, there are 27 viruses that be transmitted through the semen (Salam & Horby, 2017). Various factors affect the shedding of viruses through semen, including the immune response by the reproductive tract, alteration in the blood-testis barrier caused by inflammatory mediators, and systemic immunosuppression. The structural stability of the virus also plays an important role in viral shedding from an infected male through the semen. In appropriate protein concentrations and at ultra-low temperatures, most viruses remain viable (Gould, 1999). One such example is the influenza virus, which can be infectious after 40 years of sustenance (Merrill *et al*., 2018). Despite reports of COVID-19 transmission through the semen, there are no reports of viral cross-contamination through cryopreserved semen samples, suggesting negligible cross-contamination chances by COVID-19. However, the genetic recombination process of the coronaviruses can lead to different genotypes, causing different outbreaks (Cheng *et al*., 2007).

## Mandatory changes

All the frontline staff should use proper personal protective equipment, which includes eye protection, facemasks, gloves, shoe covers, and disposable lab coats. Based on the number of staff, facilities should arrange at least two or three mini-teams to alternate at work, thus limiting the virus spread in the event of any staff member being infected. Each team should have at least a gynecologist, a nurse, anesthesiologist, at least 2 embryologists; naming Gynecologist A, Nurse A, Anesthesiologist A, Embryologist A, and Witness A; and in the same way one should assign groups B and C. In the event that anyone in Group A (or B) comes into contact with an infected person, only the exposed group is quarantined, therefore guaranteeing the possibility to continue with the clinic’s activities. All team members should be properly instructed about safety procedures amidst this COVID-19 pandemic. Laboratory staff should minimize, and when possible entirely prevent, contact with external personnel (clinicians, nurses, obstetricians, etc.).

Avoid any contact with patients and, if not avoidable, keep 1-m distance and use proper facial protection. Train the internal personnel (security, technicians, clinicians, nurses, etc.) on how to refill the cryo-banks, thus safeguarding the cryopreserved material, and make them able to thorough inspect equipment performance under video conference with embryologists, should the lab staff be quarantined. Sanitize the environment, equipment, and devices with appropriate detergents or UV irradiation at the end of each procedure, or after each access to the workplace. Use only disinfectants with proven efficacy for cleaning procedures. All operating room staff must wear N95 masks. The clinics should document such fitting in the staff member’s file. All patients coming to the operating room should be screened 1-2 days prior to surgery via phone/portal message with standard questions and COVID-19 testing, as per local or national guidelines.

## Counseling and History Talking

Honest patient counseling and proper documentation regarding unknown effects of COVID-19 should be provided to all couples undergoing ART treatment. Face-to-face interactions should be avoided and use of telehealth technologies should be preferred (video conferencing or phone consultation). In the case where in-person consultation is necessary, one should observe proper distance; minimal attendants; facemasks, gloves and overshoes. Cycle cancellation should be discussed well in advance with patients in case of positive results, since we still do not know the effects of COVID-19 on embryos, implantation and miscarriage. Concurrently, it is important to have contingency plans in place when treatment in the ART unit is not possible, as in situations in which the whole staff is under quarantine. In this case, patients should be allowed to continue their treatment in another ART referral center. However, proper and well-documented informed consents should be signed prior starting any ART treatment (Souza *et al*., 2020).

## Staff and patient protection and availability of tests

The European Society of Human Reproduction and Embryology (ESHRE) has issued an ART Triage questionnaire, which needs to be filled by all the persons entering any ART unit, including staff (ESHRE, COVID-19 working group, 2020). The aim of filling the ART triage questionnaire is to understand any potential infection source of staff and patients visiting for ART treatment. All the potential infectious cases should be sent for COVID-19 testing according to local or national guidelines.

## Effect on gametes

### COVID-19 transmission through the Semen, Semen Collection, and Cryopreservation

In limited studies about the transmission of COVID-19 from blood to the reproductive tract in males, the results are still unclear. Systemic viral infections, like mumps-orchitis, can affect the reproductive tract in males, including the testicles. Germ cells are usually protected by the testicular immunogenic system; however, some viruses crosses the blood-testes barrier and enter the reproductive tract, resulting in a testicular immune response (Zhao *et al*., 2014). In the case of COVID-19 crossing the blood-testis barrier, in a study of twelve males with COVID-19, there was no COVID-19 transmission in semen and testicular biopsy samples (Ci *et al*., 2020), suggesting no sexual transmission of the virus. In another study assessing COVID-19 transmission through the semen in thirty-four adult COVID-19 positive males, there was COVID-19 detected in their semen; however, 19% had scrotal discomfort (Pan et al., 2020). Although this study suggests no viral transmission through the semen in infected males, this scrotal discomfort remains unclear.

### Detailed plan and recommendations for Andrology and Embryology labs

In the aforementioned studies, there was no viral transmission through either the semen or in testicular samples; however, in one recent study involving the semen of 38 positive COVID-19 males, 23 (60.5%) were clinically recovering, and 15 (39.5%) with acute infection, were tested for COVID-19 in their semen; 6 (15.8%) were positive for COVID-19; 4 in the acute stage, and 2 in the recovering group (Li *et al*., 2020). From this study, it is evident that the transmission of COVID-19 through the semen is possible, meaning that extra care needs to be taken while dealing with semen in the lab. There are no studies checking whether COVID-19 can be sexually transmitted, only time will tell. On a practical note, in an andrology laboratory, semen collection and cryopreservation are very important and routine procedures. Apart from following good laboratory techniques, andrologists need to be extra vigilant. Since reports of COVID-19 transmission through the semen, it is necessary to take proper measures in semen collection (Li *et al*., 2020). All males should be tested for COVID-19 and only those patients with negative tests should be allowed to continue their fertility treatment. In the case of semen cryopreservation, a separate tank should be assigned for all COVID-19 positive males. If possible, dedicated areas and equipment should be assigned for patients with COVID-19, followed by thorough disinfection after the procedure. All andrologists should have proper understanding on how to handle semen samples, and it is the duty of scientific directors/senior staff to properly train all staff members on a different procedure, where possible risks concerning viral transmission are high. Air quality requires special attention, with proper filtration systems in the Andrology laboratory as well. Mobile towers can be used for additional filtration of the air inside the Andrology laboratory.

There must be a person dedicated to all andrological procedures, with proper personal protective equipment (PPE) and without any outside interference in doing their job. The collection room must have proper written instructions for semen collection procedures, and person-to-person contact should be avoided. All single-use materials should be disposed off immediately after use. Positive cases should not use a Makler chamber or hemocytometer, and the slides must be discarded after use. Once the semen sample is collected and examined, the lid should be tightly closed, and all the materials should be discarded in individual bins, which should be disposed of immediately, followed by a thorough cleaning.

One should avoid freezing raw semen samples, and high-security cryo-vials should be used for sperm cryopreservation, preferably using the density gradient wash method. Also, the transportation of these cryopreserved samples between centers should be discouraged during this epidemic. A possible threat to all cryopreserved samples is the liquid nitrogen contaminated with COVID-19, which should be taken care at the time of thawing. Wherever possible, safety cabinet class II should be used, as they protect the operator in the handling of the samples. Sperm cryopreservation should be considered in a special vulnerable group of patients, due to loss of valuable time for their fertility preservation. This group of patients includes males undergoing different medical treatment for improving semen quality and quantity, or they are males with autoimmune or inflammatory diseases, and they have short fertility windows for achieving parenthood.

### Embryology Laboratory

In embryology laboratories, we need to take certain precautions in these epidemic situations for smooth working. We know that it is routine to discuss different embryological procedures between couples and embryologist; however, one should avoid in-person counseling for now; physicians should talk to patients using tele calling. It is wise to keep adequate time in-between cases for thorough cleaning. Standard quality control procedures should be followed. Mini embryologist teams (one senior and one junior) can be assigned to work in the embryology laboratory in cases of heavy workloads. So far, we do not have any study about the transmission of COVID-19 through the follicular fluid from patients who recovered from the infection. One should take extra-care when working with follicular fluids, and avoid spillage. Embryologists should make sure of proper lid closing of the follicular fluid container after oocyte screening, for immediate disposal.

As per the latest updates from the Centers for Disease Control and Prevention (CDC) in the USA and others in Europe, there is no clear evidence of any detrimental effects of COVID-19 infection on pregnancy. Nevertheless, we should bear in mind that viral infections can be more problematic during the management of positive COVID-19 pregnant women, since some of the medications used in virus-infected patients may not be recommended during pregnancy. Frozen Embryo Transfer (FET) can be postponed for women who cannot postpone their ovarian stimulation, such as women planning for anticancer therapy, poor ovarian reserve, and/or advanced maternal age.

Any risk of viral contamination to gametes or embryos in the IVF laboratory is likely to be minimal when following protocols of repeated washing, which will result in high dilution of any possible contaminants (if at all). Home collection can be ideal in cases of positive patients.

So far, there is no reliable test available to predict disease because of the high false-negative rates. As a result, all patients should be considered positive for coronavirus.

### Embryology Laboratory Suggestions during “down-time” Embryological-procedures

- Routine good laboratory practices should be followed with proper protection of staff.
- Use Quaternary ammonia compound lab disinfectants, which are tried and tested (Oosafe).
- Observe proper cleaning of safety cabinets before any procedure.
- Use a separate set of dishes for every patient before the procedure.
- The theatre, trolley, bed, and other contact equipment in the theatre must be properly sanitized before and after any procedure.
- Extra care should be taken to reduce exposure to native follicular fluid (spillage inside LAF, tight lid, separate bins, and immediate disposal).
- Increase the volume and number of flushing media droplets used for washing the gametes.
- A closed type vitrification system, which avoids direct contact of embryos to liquid nitrogen, can be used to avoid cross-contamination.
- Entire bench-top incubator/compartments should be kept for all positive or suspected cases to avoid risks of cross-contamination.
- Embryoscope can be ideal for culturing embryos of positive patients up to day 5/6 without disturbing the dish, minimizing any possible cross-contamination.
- Use separate dedicated equipment for all COVID-19 positive cases.
- Freeze-all protocols should be encouraged in the current situation.
- A separate storage tank should be kept for all positive and quarantine cases during the pandemic.

### Other precautions

- Upon entry, use a 6-step hand washing before, after and in-between all procedures.
- Follow universal precautions in suspected cases, where any ART treatment is provided, including the use of PPE kits.
- Organize the staff in mini-teams.
- Disinfect pipette holders after every procedure.
- Minimize in-person interactions of laboratory staff with patients.
- Continuously monitor liquid nitrogen tanks on a daily basis.
- Train nurses, technicians, and/or security personnel to top-up the liquid nitrogen. It should always be done under embryologist’s supervision through video calling.
- Liquid nitrogen suppliers must be informed to reserve liquid nitrogen and to avoid a possible shortage situation.
- These suppliers may be forced to prioritize the supply of oxygen to hospitals if the number of infected patient admissions increases, so having adequate backup storage in your center should also be considered.
- Keep the storage tank room temperature as low as possible to reduce the evaporation of liquid nitrogen.
- Regular back-up generator checks must be followed.
- Consider adequate diesel supply for the generator.
- Embryologists should avoid interacting with the patient or other general staff on the floor, unless it is urgent.
- Proper monitoring and precautions (removing outer packing, wiping with IVF-grade disinfectants) in the movement of media and consumable deliveries inside and outside the embryology laboratory.
- Avoid too much unnecessary opening and closing of doors inside the laboratory.
- Daily replacement of all sticky mats.
- Mobile VOC’s and pollutant removers in both Andrology and Embryology (CodaAero/Air towers).
- All staff should carry their pen, since pen sharing should be avoided in the current situation.
- Frequent door handle cleaning with gloves on should be meticulously practiced, and the gloves must be disposed of immediately after cleaning.
- Air handling units should be switched on all the time, to create positive pressure and to provide HEPA filtration, which minimizes the chances of viral spread (if at all), the size of which is much bigger than filter pores.
- Movement of embryologist through OT should be minimized as much as possible, and the pass-box/door opening should be minimized.
- Perform preventive maintenance that does not involve an outside person, to be ready for a high volume of cases after the “down-time”.

## Summary and Conclusion

 Utmost cooperation among different units and team members is compulsory for managing such crises efficiently. Last but not the least; all healthcare professionals should be given the confidence by their employees about their job not being at risk. Last, we want to express our solidarity towards the entire scientific community for their tireless efforts to find the cure for COVID-19 in the form of vaccination, and for the entire healthcare community as well as our patients for the cooperation in this unprecedented situation to overcome this pandemic.
